# 523. Use of Immune-Viral Dynamics Modeling to Understand Molnupiravir Drug Effect for COVID-19

**DOI:** 10.1093/ofid/ofab466.722

**Published:** 2021-12-04

**Authors:** Youfang Cao, Wei Gao, Ruthie Birger, Julie Stone

**Affiliations:** 1 Merck & Co. Inc, West Point, Pennsylvania; 2 Merck & Co., Inc., Kenilworth, New Jersey

## Abstract

**Background:**

Molnupiravir (MOV) is an orally administered ribonucleoside prodrug of β-D-N4-hydroxycytidine (NHC) against SARS-CoV-2. Here we present viral dynamics analysis of Phase 2 clinical virology data to inform MOV Phase 3 study design and development strategy.

**Methods:**

An Immune-Viral Dynamics Model (IVDM) was developed with mechanisms of SARS-CoV-2 infection, replication, and induced immunity, which together describe the dynamics of viral load (VL) during disease progression. Longitudinal virology data from ferret studies (Cox, et al. Nat. Microbiol 2021:6-11) were used to inform IVDM, which was further translated to human by adjusting parameter values to capture clinical data from MOVe-IN/MOVe-OUT studies. Different placements of drug effects (on viral infectivity vs. productivity) and representations of immune response were explored to identify the best ones to describe data. A simplified 95% drug effect was implemented to represent a highly effective dose of MOV.

**Results:**

IVDM showed data were best described when MOV acts on viral infectivity, consistent with the error catastrophe mechanism of action. A cascade of innate and adaptive immune response and a basal level activation enabled durable immunity and continued viral decay after treatment end. IVDM reasonably describes VL and viral titer data from animals and humans. Influence of MOV start time was explored using simulations. Consistent with the ferret studies, simulations showed when treatment is started within the first week post infection, MOV reduces viral growth, resulting in a lower and shortened duration of detectable VL. When started later (e.g. >7 days since symptom onset), the magnitude of drug effect is substantially diminished in a typical patient with an effective immune response which reduces VL prior to treatment start. Further work is needed to model response in patients with longer term infection, where MOV drug effects may have more persistent utility.

**Conclusion:**

A COVID-19 IVDM developed using multiscale MOV virology data supports drug action on viral infectivity and importance of interplay of treatment and immune response and can describe infection time course and drug effect. IVDM provided mechanistic interpretations for VL drug effect in clinical studies.

**Disclosures:**

**Youfang Cao, PhD**, Merck & Co. (Employee) **Wei Gao, PhD**, Merck & Co., Inc. (Employee, Shareholder) **Ruthie Birger, PhD**, Merck (Employee) **Julie Stone, PhD**, Merck & Co., Inc. (Employee, Shareholder)

